# Theoretical Analysis of a Microring Resonator Array with High Sensitivity and Large Dynamic Range Based on a Multi-Scale Technique

**DOI:** 10.3390/s18071987

**Published:** 2018-06-21

**Authors:** Wenqin Mo, Huiyun Liu, Fang Jin, Junlei Song, Kaifeng Dong

**Affiliations:** 1 School of Automation, China University of Geosciences, Wuhan 430074, China; jinfang78@cug.edu.cn (F.J.); songjunlei@cug.edu.cn (J.S.); dongkf1981@163.com (K.D.); 2 Hubei key Laboratory of Advanced Control and Intelligent Automation for Complex Systems, Wuhan 430074, China; 3 Department of Electronic and Electrical Engineering, University College London, London WC1E 7JE, UK; huiyun.liu@ucl.ac.uk

**Keywords:** microring resonator, optical sensors, multi-scale technique, dynamic range, finite element method

## Abstract

By using a multi-scale measurement technique, a high-sensitivity and large dynamic-range sensor array, which consisted of a single resonator and a series of cascaded resonators with a sensing ring and a reference ring, was modeled, and its transmission properties were investigated theoretically and numerically. We also set forth the principle of a multi-scale measurement technique based on the transmission spectrum of a resonator. This sensor array could have a nearly tenfold increase in sensitivity, and an improved dynamic range in an arrow wavelength range. The simulated results were in good agreement with the theoretical analysis.

## 1. Introduction

Optical microring resonators have extensive applications in wavelength filters [1], label-free biosensors [2,3,4,5], pressure sensors [6,7,8], and acceleration sensors [9,10] due to their outstanding properties of micro-size, the complementary metal-oxide-semiconductor (CMOS) compatibility and immunity to electromagnetic fields [11,12]. In sensor applications, the general sensing theory is that the change in refractive index induced by the external physical variables causes a corresponding shift in the resonant wavelength of the resonator. As sensitivity is the key performance indicator for a resonator sensor, there are multiple examples of structures constructed from various techniques [13,14,15] which were designed in an effort to improve their sensitivity. The Vernier effect [13,16,17,18], which is generated by the cascades of two ring resonators with slightly different free spectral ranges (FSRs), was theoretically and experimental proven to be a high-performance solution to improving the sensitivity of ring resonators [19]. Generally, the design and optimization of a sensor should balance its sensitivity and its dynamic range, while in other applications, such as acceleration detection, a large dynamic range and a high sensitivity are of equal importance [9]. For a resonator sensor, the FSR is directly proportional to the measurement range, and as a result, restricts its dynamic range. The cascaded microring resonators based on the Vernier effect can effectively expand the FSR [20,21]. However, simply increasing the FSR poses a serious dispersion effect on the consistency of the refractive index and the sensitivity, due to the extended broad-spectrum range. Therefore, it is desirable to undertake further study on the improvement of dynamic range, while obtaining high sensitivity in a narrow spectral range, so as to fully enhance the performances of resonator sensors.

In this work, we propose a sensor array including a single resonator and a series of cascaded resonators with a sensing ring and a reference ring. The multi-scale measurement theory and the sensing performances of the proposed sensor are discussed in detail. The theoretical analysis showed that the sensor array is expected to have a tenfold increase in sensitivity and improved dynamic range when compared with the single resonator. We investigated the properties of spectral transmission with the simulation softwares, COMSOL Multiphysics and Matlab. The simulations of the sensor array were carried out with three scales, and the results were in good agreement with the theoretical estimation. The advantage of our design was the achievement of a large sensitivity and a high dynamic range at a narrow wavelength range, which possibly reduces the effect of wavelength dispersion on the consistency of the refractive index and sensitivity.

## 2. Sensor Design and Theoretical Analysis

### 2.1. Multi-Scale Measurement Theory

The multi-scale measurement technique is a method of enhancing the sensitivity and measurement range simultaneously, which is commonly used in laser phase-shift rangefinders. It consists of two or more measurement scales, which mutually measure the same distance object. A phase-modulated laser signal with a high frequency is called a precise scale, while that with a low frequency is called a rough scale [22]. The precise scale ensures the highest sensitivity for the sensor, while the rough scale provides the maximum measurement range. In this way, the conflict between the sensitivity and the dynamic range in a single scale can be solved.

**Figure 1:**
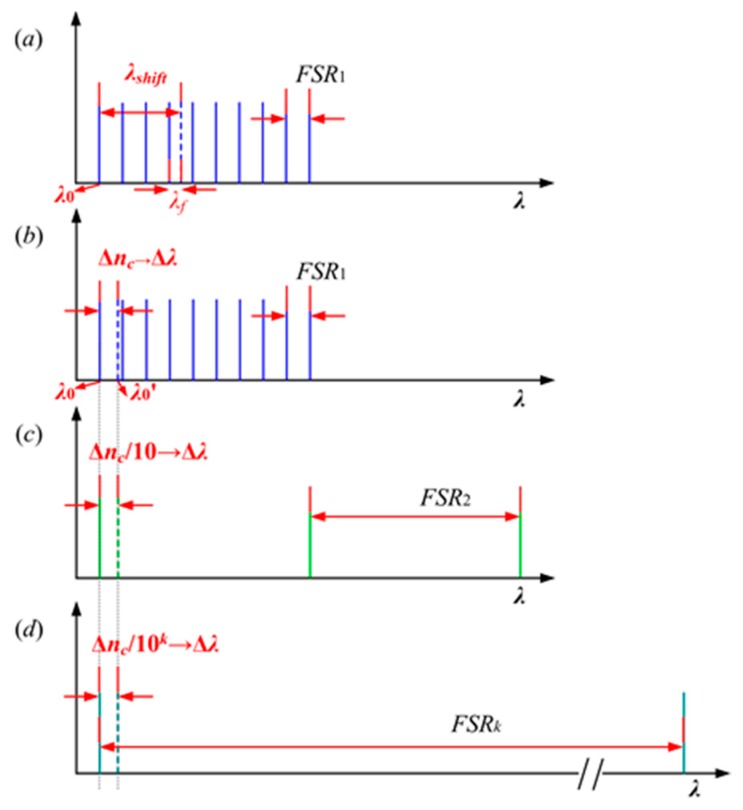
Illustration of the multi-scale measurement theory.

With respect to the resonator sensor, the shift in resonant wavelength induced by the refractive index change can be expressed as

(1) $$\lambda_{shift}=m\cdot FSR_{1}+\lambda_{f}$$

where *FSR*_1_ is free spectral range for the resonant wavelength of a single resonator, *m* is the integral number of *FSR*_1_ and is unknown, and *λ_f_* is the fraction of *FSR*_1_ determined by the wavelength shift measurement. Figure 1a indicates that if *m* does not equal zero, the equation has multiple solutions, meaning the resonant wavelength shift exceeds one *FSR*_1_. In these conditions, it is necessary to acquire both *m* and *λ_f_* in order to accurately measure the change in resonant wavelength. The ambiguity of Equation (1) can be solved through the introduction of more than one scale in a narrow wavelength range to detect the change in refractive index. The most precise scale of the sensor is called the “main scale”, which is used for the fine measurement of the wavelength shift. 

Figure 1b–d show the theory behind the multi-scale measurement. Assuming that the resonant wavelength resolution is Δ*λ*, the maximum change in refractive index under the sensitivity *S_0_* of the rough scale is Δ*n_c_*. Meanwhile, the minimum change in refractive index can be obtained as $\Delta n_{c}/10^{k}$ under the most precise scale with the sensitivity $10^{k}S_{0}$. Unlike a single pair of cascaded microrings, the maximum and minimum changes in refractive index in these conditions can be detected in a narrow wavelength range, without covering the very broad spectra generated by the Vernier effect.

### 2.2. Cascaded Microring Resonators and Sensitivity

**Figure 2:**
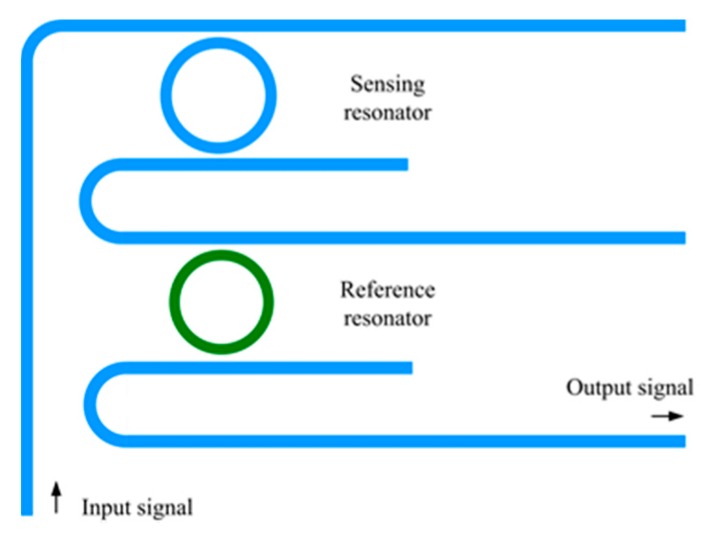
Illustration of the cascaded microring resonators.

Figure 2 illustrates the structure of both microring resonators with different circumferences cascaded in an add/drop configuration. In each pair of cascaded micorings, one served as the sensing resonator, while the other was the reference resonator. At resonance, a part of the input signal was coupled into the sensing resonator, before being coupled into the mutual straight waveguide, after travelling a half-path length of the sensing ring. The coupled signal acted as the input into the mutual straight waveguide, and was again coupled into the reference resonator, as described above.

**Figure 3:**
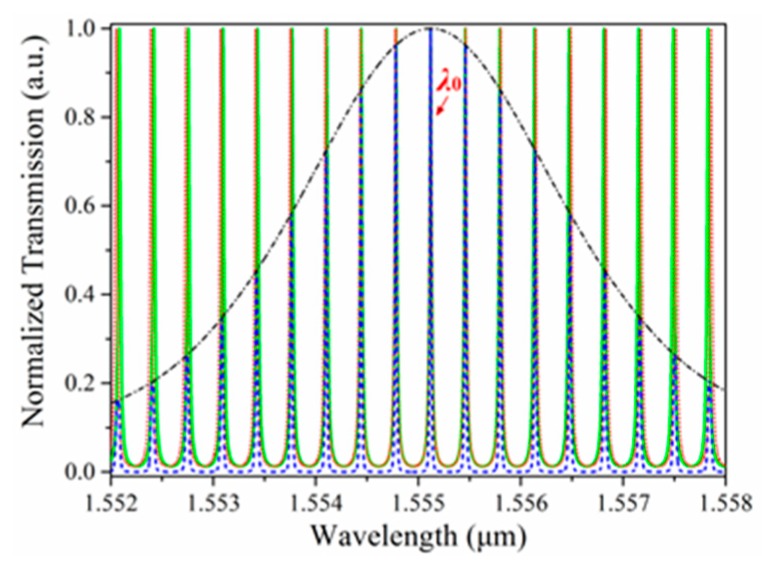
Transmission spectra of the individual sensing resonator, the reference resonator, and the cascaded resonators.

The cascaded resonators had different circumferences, and thus, different free spectral ranges. When the difference in FSR is smaller than the full-width at the half-maximum of the resonance peaks of the sensing and reference resonators, the Vernier effect takes place [13]. Figure 3 shows the transmission spectra of the individual sensing resonator (normal line), the reference resonator (dotted line), and both resonators cascaded (dashed line). Assuming two resonators happened to have spectrum peaks at the same resonant wavelength *λ*_0_, the difference in resonant wavelength between these two resonators on either side of *λ*_0_ grew wider due to the difference in FSR. The result was a reduction of the superposition in the transmission spectra of both resonators, and an envelope signal was then generated on the overlapping peaks of the cascaded resonators. In measurement, only the sensing resonator was exposed to the environmental change, while the reference resonator was covered with SiO_2_, isolated from the variation in refractive index.

**Figure 4:**
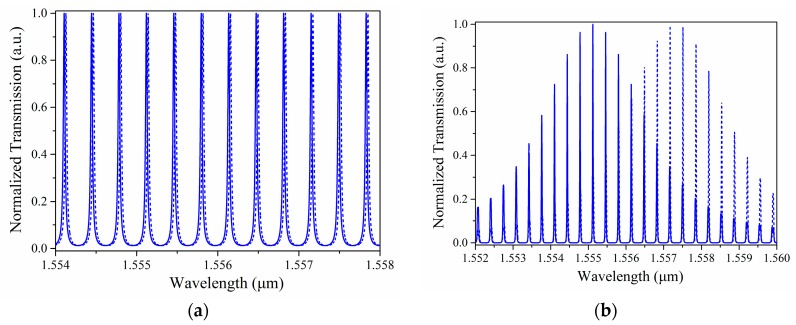
Wavelength shifts in the transmission spectra of (**a**) the individual sensing resonator, and (**b**) the cascaded resonators.

Once a sensing signal was imposed on the cascaded resonators, a variation in the refractive index or the circumference of the resonator caused a resonant wavelength shift of the sensing resonator, resulting in a change in the superposition in the transmission spectra of both resonators, and thus, a shift in the central wavelength of the envelop signal. As shown in Figure 4, when the resonant wavelength of the individual sensing resonator shifted slightly, the central wavelength of the cascaded resonators changed significantly under the influence of the same external change. Therefore, a slight change in sensing signal was easily detected by monitoring the variation in central wavelength.

Next, we determined the sensitivity of the cascaded resonators. For a single sensing resonator, the sensitivity could be given as

(2) $$S_{single}=\frac{\partial \lambda_{sen}}{\partial n_{env}}=\frac{\frac{\partial n_{eff,sen}}{\partial n_{env}}\lambda_{m}}{n_{g,sen}}$$

where $S_{n}=\partial n_{eff,sen}/\partial n_{env}$ is the change of the effective index for the sensing resonator in response to the environmental change in refractive index, and *m* is the resonant order of the microring. In Equation (2), *n_g,sen_* is the group index of the sensing resonator, as given in Reference [14],

(3) $$n_{g,sen}=n_{eff}\left(\lambda_{m}\right)-\lambda_{m}\frac{\partial n_{eff}}{\partial \lambda }$$

Based on Equations (2) and (3), the sensitivity of the single microring depended on the wavelength dispersion.

According to the Vernier effect, the sensitivity of the cascaded resonators is defined as the relationship between the central wavelength *λ_central_* of the envelop curve and the change in refractive index [13,16], and was expressed as

(4) $$S_{cascaded}=\frac{\partial \lambda_{central}}{\partial n_{env}}=\frac{FSR_{ref}}{FSR_{ref}-FSR_{sen}}\cdot \frac{\frac{\partial n_{eff,sen}}{\partial n_{env}}\lambda }{n_{g,sen}}=N\frac{\frac{\partial n_{eff,sen}}{\partial n_{env}}\lambda_{m}}{n_{g,sen}}$$

where *FSR_sen_* and *FSR_ref_* are the free spectral ranges of the corresponding resonators. From Equations (2) and (3), the sensitivity of the cascaded ring resonators was enlarged by a factor $=\frac{FSR_{ref}}{FSR_{ref}-FSR_{sen}}$. It could be inferred that a small difference in free spectral range between the cascaded resonators could greatly increase its sensitivity. However, if the system requires a large dynamic range, the very broad spectrum generated due to the Vernier effect should be monitored. As a result, the consistency of the sensitivity cannot be guaranteed in the whole spectrum induced by the Vernier effect, and the actual dynamic range is limited by the spectral range of the optical spectrum analyzer (OSA) or detectors.

**Figure 5:**
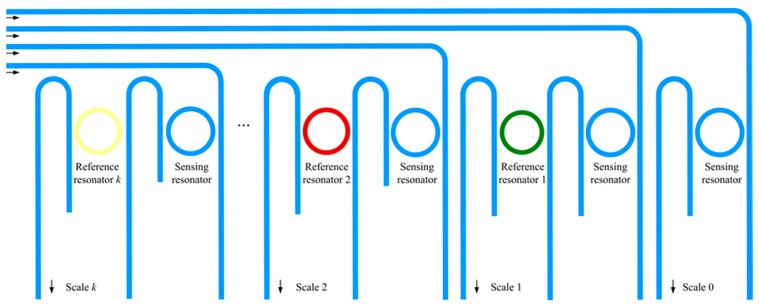
Layout of the sensor array with the multi-scale measurement.

Figure 5 demonstrates a schematic layout of our proposed sensor array based on a multi-scale measurement technique. It is reasonable to expect the sensitivity to be increased tenfold with every addition of a cascaded ring resonator, if *k* groups of cascaded resonators satisfy the individual amplification factors as

(5) $$N_{k}=10N_{k-1}=\cdots =10^{k-1}N_{1}=10^{k}N_{0}$$

where *N_k_* is the amplification factor of the cascaded resonators in group *k*(=0, 1, 2*……*), and *N*_0_ represents the amplification factor of a single sensing resonator, and is equal to 1. 

The resonant wavelengths of the cascaded sensing and reference resonators coincided at wavelength λ_0_. Considering dispersion, the next coincident resonant wavelength of both resonators [16] was expressed as

(6) $$\begin{array}{c} {\lambda_{0}}^{\prime }=\lambda_{sen,N_{k}}=\lambda_{0}+\overset{N_{k}}{\underset{m=1}{\sum }}FSR_{sen,m}\\ {\lambda_{0}}^{\prime }=\lambda_{ref,N_{k}-1}=\lambda_{0}+\overset{N_{k}-1}{\underset{m=1}{\sum }}FSR_{ref,m} \end{array}$$

where *FSR_sen,N__k_* and *FSR_ref,N__k-1_* are the free spectral ranges of the sensing and reference resonators at wavelengths *λ_sen,N__k_* and *λ_ref,N__k-1_*, respectively. By substituting an average free spectral range of both resonators between *λ*_0_ and *λ*_0_’ in Equation (6), the amplification factor, *N_k_*, could be expressed as

(7) $$N_{k}=\frac{\bar{FSR_{ref}}}{\bar{FSR_{ref}-FSR_{sen}}}$$

Here, the reference resonator in the “main scale” had the maximum amplification factor, *N_k_*, and as a result, provided the maximum sensitivity to the sensor array. Using a specific measurement scale, we measured the central wavelength shift in the periodical envelope signal to determine the corresponding change in refractive index. 

Once the *FSR_sen_* was determined, the average free spectral range of the reference resonators could be calculated using Equations (5) and (7). Moreover, the free spectral range of the resonator [14] was defined as

(8) $$FSR=\frac{\lambda_{m}^{2}}{Ln_{g}}$$

From the above equation, the circumferences of the sensing and reference resonators for each pair in the sensor array could be calculated.

### 2.3. Detection Limit and Dynamic Range

Generally, the dynamic range of a refractive index sensor is defined as

(9) $$\mathrm{DR}\left(\mathrm{dB}\right)=10\;\log_{10}\frac{∆n_{max}}{LOD}$$

where Δ*n_max_* and *LOD* represent the sensor’s maximum detectable signal and the detection limit, respectively. For a single resonator, FSR poses a limitation to the maximum detectable wavelength [13]. In a practical measurement, *LOD* mainly depends on the wavelength detection limit and sensitivity, as described in Reference [23],

(10) $$LOD=\frac{\delta \lambda }{S}$$

According to References [23,24], the wavelength detection limit *δλ* could be estimated by

(11) $$\delta \lambda =\sqrt{\sigma_{1}^{2}+\sigma_{2}^{2}+\sigma_{3}^{2}}$$

Here, *σ*_1_ is the standard deviation of the fitting curve of the peak spectral signal, which is aimed to accurately determine the resonant wavelength for a single resonator, and the central wavelength for the cascaded resonators. *σ*_2_ and *σ*_3_ are the standard deviations due to the temperature noise and the spectral resolution, respectively. 

From Equations (9) and (10), the dynamic range of the sensor array could be expressed as

(12) $$\mathrm{DR}\left(\mathrm{dB}\right)=10\;\log_{10}\frac{∆n_{max}}{\delta \lambda /N_{k}S_{0}}=10\;\log_{10}\frac{∆n_{max}}{\delta \lambda /S_{0}}+10\;\log_{10}N_{k}$$

Based on the above theoretical analysis, the precision of the sensor array was decided by the choice of the “main scale” resonator, which provided the maximum sensitivity. Furthermore, the maximum signal was dependent on the measurement range of the first sensing resonator, as shown in Figure 5. Here, the sensitivity of the “main scale” was increased by the factor *N_k_* (*N_k_* = 10*^k^N*_0_) through the group of cascaded resonators, *k*. In the case of identical wavelength detection limit *δλ* for both the single resonator and the proposed sensor array, the detection limit of the main scale was *δλ*/*N_k_S*_0_. Consequently, the dynamic range of the sensor array could be theoretically improved by a value of 10k dB when compared with the single resonator.

## 3. Numerical Validations of Optical Transmissions

### 3.1. Simulation Setting

This section introduces the simulation setting of the sensor array. According to the relationship between an average value of *FSR_sen_* and *FSR_ref_*, described in Equation (7), the design parameters of the cascaded resonators are listed in Table 1. The effective indices of the silicon-on-insulator (SOI) waveguide were obtained as a function of wavelength, using the COMSOL Multiphysics software. The transmission spectra of the three scales were calculated in the Matlab software, with the consideration of waveguide dispersion.

### 3.2. Simulation Results

**Figure 6:**
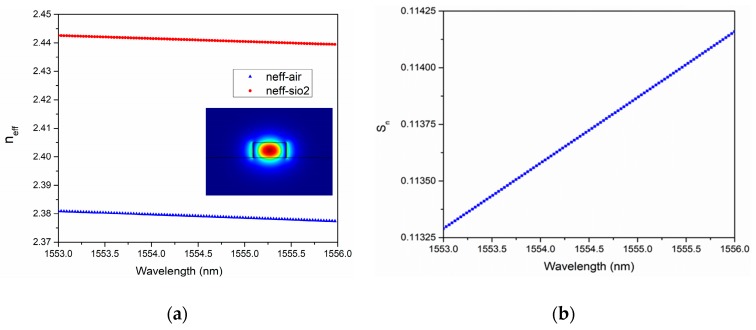
Waveguide dispersion of the (**a**) effective index, and (**b**) the effective sensitivity (*Sn*) for the waveguide on SOI.

We investigated the influence of wavelength dispersion on the waveguide for the SOI wafer using the COMSOL Multiphysics software with a finite element method. Figure 6a shows the dispersion of the effective indices of the quasi-transverse-electric (quasi-TE) mode for the sensing and reference resonators. Figure 6b depicts the effective index sensitivity (*S_n_*) independence on the wavelength, in the case of the air upper cladding. The effective index and its sensitivity changed relatively slightly upon a variation of the detectable wavelength in a narrow wavelength range. For the sensing resonator, a group index was calculated as 4.2224, while it was calculated as 4.0577 for the reference resonator covered by SiO_2_.

**Figure 7:**
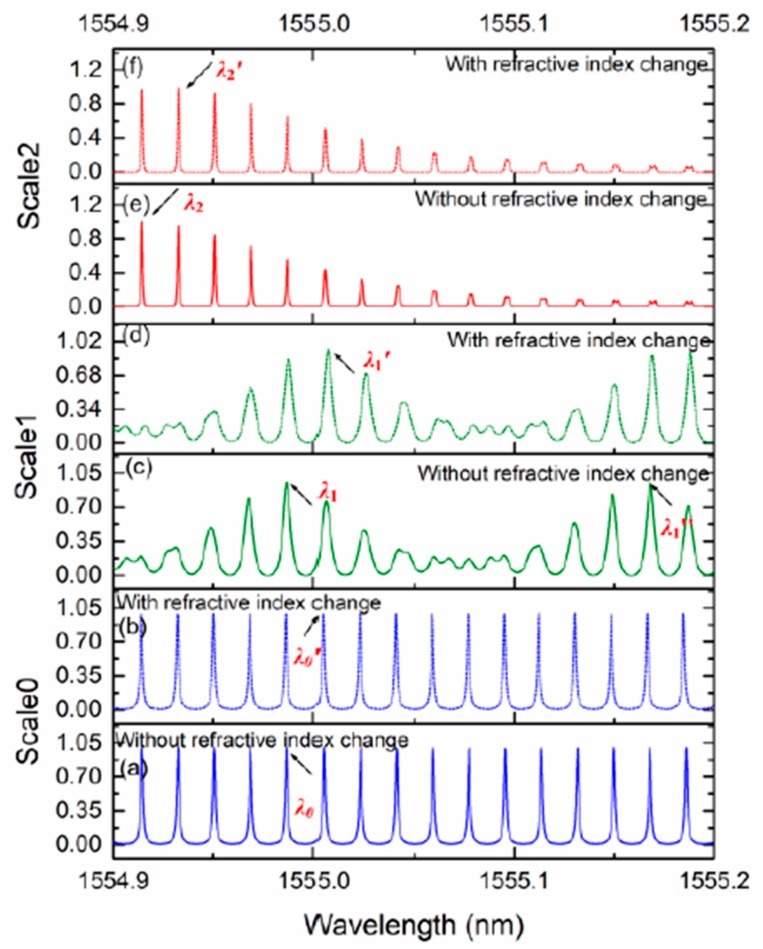
(**a**) Normalized transmission spectra of Scale 0 without (normal line) and (**b**) with a change in refractive index (dashed line). (**c**) Normalized transmission spectra of Scale 1 without (normal line) and (**d**) with a change in refractive index (dashed line). (**e**) Normalized transmission spectra of Scale 2 without (normal line) and (**f**) with a change in refractive index (dashed line).

Figure 7 shows that the simulated transmission spectra for three pairs of cascaded resonators with their respective theoretical sensitivities of *S*_0_, *S*_1_(=10*S*_0_), and *S*_2_(=100*S*_0_). The output signals were normalized to the intensity of the incident light. Since the maximum spectral shift could not exceed one FSR, the transmission spectrum of a single sensing ring was simulated considering the change in the upper cladding refractive index of 4 × 10^−4^ RIU (refractive index unit). It shows a shift in the resonant wavelength from *λ*_0_ to *λ*_0_’. In the case of Scale 1, both resonances for the sensing and reference rings coincided at wavelength λ_1_. When the change in refractive index was 4 × 10^−5^ RIU, the central wavelength of Scale 1 varied from *λ*_1_ to *λ*_1_’. At *λ*_1_’’ = 1555.1681 nm, both resonances of the sensing and reference rings in Scale 1 coincided again. When the variation in refractive index was 4 × 10^-6^ RIU, the central wavelength of Scale 2 shifted from λ_2_ to λ_2_’. Therefore, the working wavelength range for the sensor array lay between λ_2_ and λ_1_’’, around 260 pm. If the FSR of the single sensing ring was around 18 pm, the FSR of Scale 2 was approximately 1800 pm, according to the relationship between sensitivity and FSR, determined from Equations (5) and (7). That is to say, in the case of a typical sensing structure based on the Vernier effect, the central wavelength of the envelope signal should be monitored throughout almost the whole spectral range of around 1800 pm in an effort to obtain the same dynamic range of the sensor array. It is evident that the working wavelength range was greatly reduced using the multi-scale measurement technique. 

It is worth noting that the difference between *FSR_sen_* and *FSR_ref_* should be smaller than the value of the full-width at the half-maximum of the resonance peaks of both resonators in each scale. For this example, the coupling coefficient between the straight waveguide and the ring resonator should be chosen appropriately according to the relationship between *FSR_sen_* and *FSR_ref_* for Scale 1 and Scale 2. In this case, the power coupling coefficients for Scale 1 and Scale 2 were *k*_1_ = 0.2 and *k*_2_ = 0.64, respectively. 

The simulated sensing performances of the three scales are listed in Table 2. The central wavelength of the transmission peaks was determined using the position of the calculated maximum of the enveloping curve. The standard deviation with a Lorentzian fit for the enveloping curve was assumed as 10 pm for all scales. In the case of a sensor on an SOI wafer, and an optical spectrum analyzer with a spectral resolution of 1 pm, the deviations σ_2_ and σ_3_ are negligibly small when compared with standard deviation *σ*_1_ [24]. From the simulated value of the resonant wavelength shift in Table 2, the sensitivities of Scale 2 and Scale 1 were calculated based on Equation (4). It is evident that the sensitivities of Scale 2 and Scale 1 were improved by almost 102.55 times and 10.14 times, respectively, when compared with a single resonator. Moreover, on the basis of Equation (12), the calculated dynamic range improvement is also listed in Table 2. When compared with the single resonator, the dynamic range was theoretically improved by nearly 10k dB in this case. 

## 4. Conclusion

In this paper, we presented a resonator sensor array with a high sensitivity and a large dynamic range through the application of a multi-scale technique. The multi-scale theory and the characteristics of the sensor array were studied in detail. Theoretically, the sensor array had a tenfold increase in sensitivity, and an improved dynamic range. The optical transmission spectra and the sensing performances of the proposed structure were analyzed using the simulation softwares, COMOSL Multiphysics and Matlab. The simulated results of the sensor array with the three scales proved that a large dynamic range with high sensitivity could be achieved at a narrow wavelength range when compared with a single pair of cascaded resonators. This study possibly provides a useful guideline for the design of pressure or acceleration sensors based on ring resonators, which have a high demand for sensitivity and dynamic range.

## Figures and Tables

**Table 1:** Design parameters of the cascaded resonators.

Parameter	Symbol	Value
Core width	*W_core_*	0.5 μm
Core height	*H_core_*	0.22 μm
Refractive index of core (Si)	*n*_1_	3.476
Refractive index of upper and bottom cladding (SiO_2_)	*n*_2_	1.445
Refractive index of upper cladding (Air)	*n_air_*	1.0
Free spectral range (FSR) of Sensing Resonator	*FSR_sen_*	18.01 pm
FSR of Reference Resonator 1	*FSR_ref,_*_1_	20.01 pm
FSR of Reference Resonator 2	*FSR_ref,_*_2_	18.19 pm

**Table 2:** Performances of the sensor array.

Type	Initial Wavelength (nm)	Resonant Wavelength Shift (pm)	Sensitivity (nm/RIU)	Dynamic Range Improvement (dB)
Scale 0	1554.9868	17.75	44.37	-
Scale 1	1554.9866	18.01	450.23	10.06
Scale 2	1554.9144	18.20	4550	20.11
